# Room-temperature stable loop-mediated isothermal amplification (LAMP) reagents to detect leptospiral DNA

**DOI:** 10.2478/abm-2021-0023

**Published:** 2021-08-20

**Authors:** Pui-Yuei Lee, Yien-Ping Wong, Shuhaidah Othman, Hui-Yee Chee

**Affiliations:** Department of Medical Microbiology and Parasitology, Faculty of Medicine and Health Sciences, Universiti Putra Malaysia, 43400 Serdang, Selangor, Malaysia

**Keywords:** leptospirosis, LAMP assay, temperature, room, stabilizer, sucrose

## Abstract

**Background:**

Loop-mediated isothermal amplification (LAMP) is one of the most promising tools for rapidly detecting *Leptospira* spp. However, LAMP is hampered by cold storage to maintain the enzymatic activity of *Bst* DNA polymerase.

**Objective:**

To overcome the drawback of cold storage requirement for LAMP reagents we modified the reagents by adding sucrose as stabilizer. We then sought to determine the stability at room temperature of the premixed LAMP reagents containing sucrose.

**Method:**

Premixed LAMP reagents with sucrose and without sucrose were prepared. The prepared mixtures were stored at room temperature for up to 60 days, and were subjected to LAMP reactions at various intervals using rat kidney samples to detect leptospiral DNA.

**Results:**

The premixed LAMP reagents with sucrose remained stable for 45 days while sucrose-free premixed LAMP reagents showed no amplification from day 1 of storage at room temperature up to day 14.

**Conclusion:**

The LAMP reagent system can be refined by using sucrose as stabilizer, thus allowing their storage at room temperature without the need for cold storage. The modified method enables greater feasibility of LAMP for field surveillance and epidemiology in resource-limited settings.

Nucleic acid amplification is regarded as one of the most valued methods for assay requirements ranging from biotechnology to diagnosis of infectious diseases [1]. For decades, nucleic acid amplification has played a substantial role in addressing the challenges of disease diagnosis. Leptospirosis is a zoonotic disease in which rats are regarded as the major reservoir of leptospirosis infection because they usually remain as permanent bacterial carriers [2]. In Malaysia, leptospirosis outbreaks are often reported following heavy rainfall in affected areas. The rainfall tends to wash out rat holes, introducing leptospiral bacteria to water bodies and the soil surface, resulting in contamination of water and soil, which serve as a transmission route to humans. Despite substantial efforts to control and prevent disease outbreaks, leptospirosis infections continue to cause deaths and high morbidity among human populations [3].

Disease surveillance of leptospirosis through early detection of leptospire in rats in disease hot-spots is crucial to minimize the risk of human infection and to prevent disease outbreaks. Khairani-Bejo et al. reported the presence of leptospiral bacteria in rats captured in a residential area of Serdang, Selangor, Malaysia, and their study demonstrated that polymerase chain reaction (PCR) has better sensitivity to detect leptospiral bacteria in rats than the usual methods of culture and microscopic agglutination (MAT), which are more time-consuming and laborious [4]. Benacer et al. trapped 300 urban rats at 3 sites in Kuala Lumpur found that 20, mostly from the Chow Kit Market in Kuala Lumpur, Malaysia were positive for *Leptospira* spp. and identified isolates by culture and PCR [5]. The nucleic acid amplification method using PCR is a useful tool with which to detect *Leptospira* spp. in rats; however, PCR may have lower sensitivity than other molecular methods such as loop-mediated isothermal amplification (LAMP) [6, 7]. Additionally, PCR requires the use of a PCR thermocycler, which is not practical for routine use in resource-limited areas [6]. As such, is highly desirable to formulate an alternative method that does not require specialized equipment while allowing sensitive detection of *Leptospira* spp.

In 2000, a novel nucleic acid assay method named LAMP was first described to amplify millions of copies of bacterial DNA in a single tube in less than an hour with high specificity and sensitivity [8]. LAMP is based on an autocycling strand-displacement reaction by using a set of 4–6 oligonucleotides that recognize 6–8 DNA sequences within their target genomic region and form a loop-structured amplicon. Loop primers may also be used to improve amplification. Crucially, LAMP is amplified at a constant temperature within the range of 60–65 °C [1]. Thereby, LAMP overcomes the limitation of PCR of the need for specialized equipment [8, 9]. LAMP has the potential to detect nucleic acid of *Leptospira* spp. in humans and animals [6, 10]. Despite the advantages of LAMP, its existing protocol has limitations, including the cold chain transportation and storage of LAMP reagents [9]. *Bst* DNA polymerase must be stored at −20 °C to maintain its enzymatic activity, thereby limiting LAMP application to field surveillance and epidemiology, particularly in resource-limited regions.

Several advances toward the application of LAMP as a point-of-care detection method have been reported including electric LAMP, lyophilized LAMP, lateral flow assay LAMP, micro-LAMP, and multiplex LAMP. Lyophilized LAMP is used to describe the form of LAMP reagents processed by freeze-drying the reagents to allow their storage at room temperature [1]. Foo et al. reported development of a thermo-stabilized triplex LAMP assay with addition of raffinose as stabilizer before freeze-drying the reagents, thus eliminating the need for cold storage [11]. However, this method is not a good alternative because it is tedious and requires certain expertise of handling the operation of the freeze-dryer.

The nonreducing disaccharide sucrose provides thermal protection to *Taq* polymerase in PCR reactions [12]. However, the use sucrose as stabilizer in LAMP assays is limited despite various studies that found sugars can act to protect protein, thus avoiding loss of enzymatic activity [13]. The present study aimed to investigate the stability of a premixed LAMP reaction mixture with addition of sucrose stored at room temperature for the detection of leptospiral DNA from rat kidney.

## Methods

### Primers

A set of 6 primers designed in a previous study [10] was utilized to perform the LAMP assay by targeting a 276 bp DNA fragment spanning the *Leptospira secY*. The genomic sequence of *Leptospira interrogans* serovar Pomona SecY (*secY*), partial cds (GenBank: EU358013.1) was accessed from the NCBI Nucleotide Database (https://www.ncbi.nlm.nih.gov/nucleotide/). To identify conserved region within *secY*, multiple sequence alignment was conducted using Clustal Omega (https://www.ebi.ac.uk/Tools/msa/clustalo/). A primer set targeting the entire *secY* sequence was designed using NCBI Primer BLAST. LAMP primer sets were designed using the OptiGene LAMP Designer software (http://www.optigene.co.uk/lamp-designer/). Default parameters were used for the primer design. The LAMP primer set comprises the 2 outermost primers (F3 and B3), 2 inner primers (FIP and BIP), and 2 loop primers (LF and LB) (**Table 1**). All of the primers used in this study were synthesized by Integrated DNA Technologies Inc. (Singapore).

**Table 1:** LAMP primer sequences for *secY* of *Leptospira*

**Primer**	**Sequence (5′ – 3′)**
F3	CTTGTTCCTGCCCTTCAAA
B3	TTCGGTGATCTGTTCTCCT
FIP	TTCCGTGCCGGTAGACCA-GAACCGTAATTCTTTGTGCG
BIP	CTTGAGCCTGCGCGTTAC-AATGAGAAGAACGGTTCCG
LF	GCGAGTTGGATCACTGCTA
LB	CCGGGCTTAATCAATTCTTCTG

The genomic sequence of *Leptospira interrogans* serovar Pomona SecY (*secY*), partial cds (GenBank: EU358013.1) was accessed from the NCBI Nucleotide Database (available at: https://www.ncbi.nlm.nih.gov/nucleotide/)

LAMP, loop-mediated isothermal amplification.

### Samples

The utilization of rat kidney samples in this study was approved by the animal ethics committee of Universiti Kebangsaan Malaysia (UKMAEC) with the reference number FST/2016/AR-CAT2 and complied with national and international guidelines for the use of animals in research including the U.S. Animal Welfare Act (Public Law 89–554, 1966) including any amendments passed to 2008 as described in the USDA Blue Book (2020) including field research as described in the OLAW Institutional Animal Care and Use Committee Guidebook (2nd edition 2002), did not endanger any small or declining animal populations, and was compliant with the Convention on Biological Diversity and IUCN policy statement. Reporting follows ARRIVE 2.0 guidelines for animal research [14]. Rats (*Rattus* spp.) had been captured in selected sites of urban, semi-urban, and forest sites in Selangor, Malaysia where leptospirosis cases were reported [15]. The selected rat kidney samples were the same as those collected previously [15] and tested with a previously developed LAMP method [10]. In the present study, we used 10 rat kidney samples confirmed positive for leptospiral DNA and 10 rat kidney samples confirmed negative for leptospiral DNA. Genomic DNA from rat kidneys was extracted using a FavorPrep Tissue Genomic DNA Extraction Mini Kit (Favorgen Biotech, Taiwan). The extraction was performed in accordance with the procedures specified by the manufacturer. The concentration and purity of the extracted DNA were quantified using a NanoDrop ND-1000 spectrophotometer (Thermo Fisher Scientific) and the samples were kept at −20 °C until further use.

### Preparation of 20% sucrose

The preparation of premixed LAMP reagents was adapted from Foo et al., with modifications to avoid the use of a freeze-drying process and changes to the type of sugar used [11]. The selection of sucrose to test for its applicability in LAMP was adapted from Chen et al. [9]. Thus, 20 g of sucrose (CAS 57-50-1, molecular biology grade, catalog No. S0389, Sigma-Aldrich) was weighed and mixed with 100 mL of nuclease-free water to make 20% (w/v) sucrose.

### Preparation of premixed LAMP reagent

The 2× premixed LAMP reagents were comprised of 2× ThermoPol buffer (New England Biolabs), 12 mM of MgSO_4_ (New England Biolabs), 2.8 mM dNTPs (First Base Laboratories, Malaysia), 0.8 M betaine (Sigma-Aldrich), 8% (w/v) sucrose, 8 units of *Bst* DNA polymerase (large fragment; New England Biolabs), and nuclease-free water. This mixture was stored at room temperature up to 60 days and the LAMP reaction was performed on day 1, 3, 7, 14, 35, 45, and 60 of storage. Another set of 2× premixed LAMP reaction without sucrose was used as a control.

### Premixed LAMP reaction

Briefly, 25 μL of the LAMP reaction mixture contained 12.5 μL of 2× premixed LAMP reagents, 1 μL of calcein dye (Nacalai Tesque, Japan, CAS1461-15-0, product No. 06713-61; 1.25 mM calcein dye and 12.5 mM MnCl_2_), 5 pmol each of F3 and B3 primers, 40 pmol each of FIP and BIP primers, 20 pmol each of the LF and LB primers, various volumes of the samples (300 ng of DNA), and nuclease-free water. A positive control template containing *Leptospira secY* plasmid and nontemplate control (NC) containing nuclease-free water was included in each test. The mixture was incubated at 65 °C for 30 min for amplification and then at 80 °C for 3 min to terminate the reaction. LAMP products were visualized based on color change, fluorescence emission, and electrophoresis in 1.5% agarose gel (5 mm thick × 107 mm long) in 40 mM Tris, 20 mM acetate, 1 mM EDTA (TEA 50×; ThermoFischer Scientific, molecular biology grade, catalog No. B49) using an MT-108 Mini Horizontal Gel Electrophoresis System (Major Science) at 90 V (8.4 V/cm) for 45 min, stained with 0.5 μg/mL ethidium bromide for 10 min, and followed by destaining with distilled water for 10 min. Products in the gel were visualized at 254 nm using a ultraviolet (UV) Transilluminator 2000 (Bio-Rad).

## Results and Discussion

LAMP represents an attractive approach for rapid and deployable pathogen detection. Since its development by Notomi et al. [8], LAMP has been widely used as a molecular detection tool. One of the most outstanding advantages of LAMP is its speed. For instance, LAMP results can be obtained within 30 min, whereas PCR, which is one of the most widely used molecular detection tools, would require at least 90 min to obtain a result. However, the most critical advantage of LAMP is its simplicity whereby LAMP runs under isothermal conditions, and the method requires only a water bath or heating block [1].

To date, the usage of LAMP has been widely reported and most studies have highlighted its advantage of being rapid, while capable of detecting pathogens with high sensitivity and specificity [7, 8]. Due to its advantages, LAMP is particularly useful in epidemics as a rapid diagnostic tool to play a vital role in outbreak management. For example, during the current coronavirus disease 2019 (COVID-19) pandemic, LAMP has been deployed as molecular tool to detect and contain the spread of COVID-19 [16]. Similarly, LAMP may be used as a method to detect and control the spread of leptospirosis. Unfortunately, the requirement of cold storage of LAMP reagents limits the application of the method, especially in resource-limited settings where cold storage facilities are limited [7].

Alternatively, researchers have opted for lyophilized LAMP to eliminate the use of cold storage. Engku Nur Syafiraha et al. indicated that lyophilization of LAMP reagents with addition of sugar in LAMP enabled the reagent to maintain functionality and stability at room temperature [7]. However, a drawback of lyophilization lies in the requirement for sophisticated equipment such as a rotary or manifold freeze-dryer. The use of a freeze-dryer may alter material structure, which could affect the sensitivity and specificity of the test. Moreover, freeze-dryers are expensive and not readily available in many laboratories or in resource-limited settings [13].

Othman et al. developed and optimized a LAMP protocol using clinical and animal samples targeting the same *secY* gene region. The sensitivity of the developed LAMP system was found to be as low as 2 × 10^4^ copies of genomic DNA per reaction [10]. Despite the high sensitivity reported in the previous study, the method is not appropriate if the test was carried out in less-equipped laboratory settings due to the restriction of cold storage for LAMP reagents. Therefore, the present study modified the developed LAMP system with addition of sucrose to eliminate the need of cold storage for LAMP reagents. The 20 rat kidney samples used in the present study were the same as those tested with the LAMP method developed in previous studies [10, 15].

**Figure 1:**
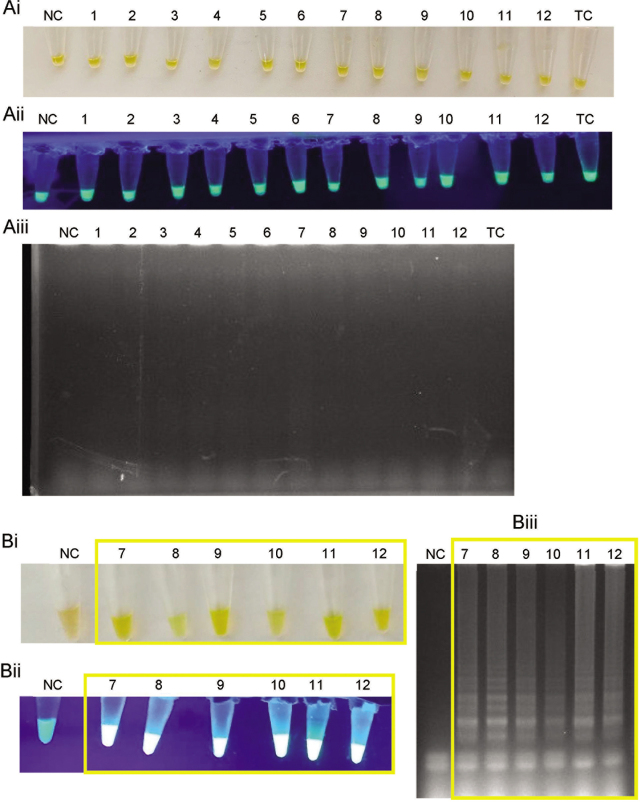
LAMP endpoint product on day 1 with the premixed LAMP reagents stored at room temperature. **(A)** Without sugar. **(B)** With 4% (w/v) sucrose (final concentration, storage at 8%). The following apply to **(A)** and **(B)**. 1–6: Rat kidney samples confirmed for negative leptospiral DNA. 7–12: Rat kidney samples confirmed positive for leptospiral DNA. **(i)** Colorimetric observation based on the change in the color of calcein dye. **(ii)** Fluorescence emission under UV light. **(iii)** Electrophoresis of LAMP products in 1.5% agarose gel at 90 V for 45 min with ethidium bromide staining. Yellow boxes indicate positive LAMP amplification where calcein dye changes from orange to green, emission of fluorescence, and ladder-like bands. Products in the gel are visualized at 254 nm using a UV Transilluminator 2000 (Bio-Rad). A positive amplification in LAMP is indicated by the formation of ladder-like bands, due to the working principle of LAMP. No size marker was used on the gel as identification of a single discrete band is not possible with LAMP. All images are representative of at least 3 replicate experiments. LAMP, loop-mediated isothermal amplification; NC, nontemplate control; TC, template control; UV, ultraviolet.

**Figure 2:**
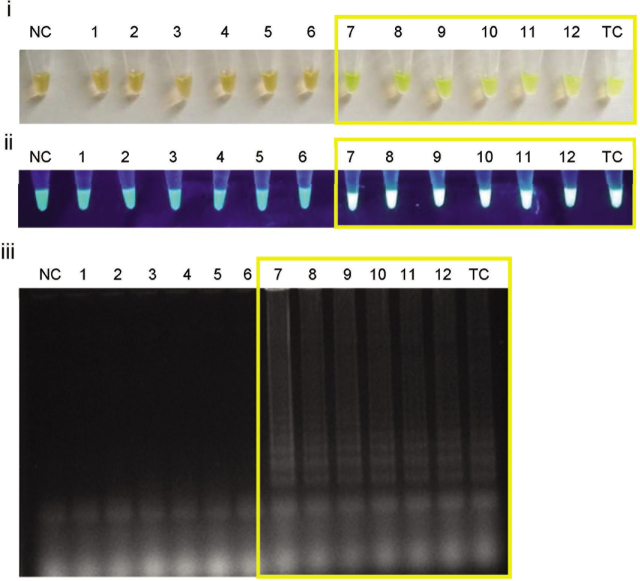
LAMP products on day 45 with the premixed LAMP reagent supplemented with sucrose and stored at room temperature. The following apply to all panels; 1–6: Rat kidney samples confirmed for negative leptospiral DNA. 7–12: Rat kidney samples confirmed positive for leptospiral DNA. **(i)** Colorimetric observation based on the change in the color of calcein dye. **(ii)** Fluorescence emission under UV light. **(iii)** Electrophoresis of LAMP products in 1.5% agarose gel at 90 V for 45 min with ethidium bromide staining. Yellow boxes indicate positive LAMP amplification where calcein dye changes from orange to yellow–green, emission of fluorescence, and ladder-like bands. Products in the gel are visualized at 254 nm using a UV Transilluminator 2000 (Bio-Rad). A positive amplification in LAMP is indicated by the formation of ladder-like bands. No size marker was used on the gel as identification of a single discrete band is not possible with LAMP. All images are representative of at least 3 replicate experiments. LAMP, loop-mediated isothermal amplification; NC, nontemplate control; TC, Template control; UV, ultraviolet.

The LAMP reaction using the sucrose-free premixed reagents stored at room temperature showed no amplification in all rat kidney samples on day 1 (**Figure 1A**). The test was conducted only for up to 14 days because it consistently showed no amplification, which presumably was because the activity of the *Bst* DNA polymerase had been lost in the sugar-free premixed reagents. Conversely, amplification was observed in the rat kidney samples that contain leptospiral DNA using the premixed LAMP reagents with sucrose stored at room temperature on day 1 (**Figure 1B**). Moreover, we found that the premixed LAMP reagents with sucrose remained stable for 45 days at room temperature (**Figure 2**). The positive samples showed changes in the calcein dye color from orange to yellow–green under visible light and emitted a fluorescent signal under UV light. For the sample known to be negative, the calcein dye remained orange, and no emission of fluorescence was observed. LAMP was also analyzed by agarose gel electrophoresis to confirm the positive samples showed ladder-like bands on the gel.

To date, studies on the use of sugar in LAMP reagents remain limited. Most of the studies reported have used treha-lose as stabilizer for the LAMP reagents because trehalose has been reported to provide a good stability for proteins in room temperature storage [7, 9]. However, others indicated that sucrose tends to provide better stability to maintain the enzymatic activity than trehalose [17]. Foo et al. adapted the preparation of premixed LAMP reagents with minor modifications of the use of a freeze-drying process and the type of sugar [11]. Meanwhile, the selection of sucrose was inspired by the study by Chen et al. to test for its applicability in the LAMP method [9]. Sucrose has been used widely to preserve enzymatic activity because it provides good stabilizing effects and the recovery of glucose-6-phosphate dehydrogenase activity in its presence is 94%–98% [18]. Sucrose can tolerate various temperature conditions and resist dehydration, while allowing reagents to be stored at room temperature [19]. Louwrier and Valk described that sucrose can maintain the enzymatic activity of *Taq* DNA polymerase for longer than reducing sugars such as glucose, raffinose, or maltose [12].

Although the exact mechanism of thermal protection by sucrose remains unclear, it is presumed that the possible mechanism of sugar in preserving the protein is due to a “glassy dynamics” hypothesis in which the “sugar forms a rigid, inert matrix in which the protein is molecularly dispersed, and the limited mobility in the glassy matrix dampens the protein mobility necessary for movement along the degradation pathway” [17]. As a result, degradation of protein is reduced by its kinetic control. In the present study, we found that sucrose maintained the enzymatic activity of the *Bst* DNA polymerase during storage at room temperature for at least 45 days.

Detection of leptospiral DNA in rat kidney samples using the room-temperature stable premixed LAMP reagents suggests that the method may also be applied to detect leptospiral DNA in clinical samples. The modification introduced in the present study would enhance the original LAMP method [10] attributes of ease-of-operation, minimal training requirements, and cost-effectiveness, thereby enhancing its potential as a point-of-care-testing method. The elimination of cold storage and machine dependence would allow the modified LAMP to be used on site to detect *Leptospira* spp. in potential outbreak areas or field surveillance where resources are limited. This would facilitate the epidemiology of leptospirosis and implementation of prevention and control strategies, particularly in the control of the reservoir animal.

There are some limitations to the present study. Various concentrations of sucrose were not tested. Thus, we are uncertain whether another concentration would provide better stability of LAMP reagents. The present study only tested sucrose. Further studies that test other concentrations of sucrose and types of sugar to optimize conditions to preserve activity of *Bst* DNA polymerase may be warranted.

## Conclusion

This improved method addresses the limitations of cold storage requirements for LAMP reagents. The premixed LAMP reagents with sucrose as stabilizer could maintain the *Bst* DNA polymerase activity at room temperature for at least 45 days. The formulation of room-temperature stable premixed LAMP reagents for the rapid detection of leptospiral bacteria may facilitate the early detection and control of leptospirosis, and enable preventive actions to be undertaken when a leptospirosis outbreak is anticipated, particularly in resource-limited settings and under field conditions.
